# *FABP2* Ala54Thr Polymorphism and Post-Training Changes of Body Composition and Biochemical Parameters in Caucasian Women

**DOI:** 10.3390/genes12070954

**Published:** 2021-06-22

**Authors:** Agata Leońska-Duniec, Katarzyna Świtała, Ildus I. Ahmetov, Craig Pickering, Myosotis Massidda, Maciej Buryta, Andrzej Mastalerz, Ewelina Maculewicz

**Affiliations:** 1 Faculty of Physical Education, Gdansk University of Physical Education and Sport, 80-336 Gdansk, Poland; katarzyna.switala@awf.gda.pl; 2 Laboratory of Molecular Genetics, Kazan State Medical University, 420012 Kazan, Russia; genoterra@mail.ru; 3 Department of Physical Education, Plekhanov Russian University of Economics, 117997 Moscow, Russia; 4 Institute of Coaching and Performance, School of Sport and Wellbeing, University of Central Lancashire, Preston PR1 2HE, UK; craigpickering1014@hotmail.com; 5 Department of Life and Environmental Sciences, University of Cagliari, 09124 Cagliari, Italy; myosotis.massidda@gmail.com; 6 Institute of Physical Culture Sciences, University of Szczecin, 70-453 Szczecin, Poland; maciej.buryta@usz.edu.pl; 7 Faculty of Physical Education, Jozef Pilsudski University of Physical Education in Warsaw, 00-968 Warsaw, Poland; andrzej.mastalerz@awf.edu.pl (A.M.); ewelina.jask@gmail.com (E.M.)

**Keywords:** sport genetics, physical activity, *FABP2* gene, polymorphism, body composition, lipid profile, adaptation

## Abstract

The functional *FABP2* Ala54Thr polymorphism (rs1799883) is strongly associated with lipid and carbohydrate metabolism, although the function of its potential modifying effect on training-induced changes in obesity-related parameters is still unknown. The aim of the present study was to investigate the influence of the Ala54Thr polymorphism on post-training changes of selected body mass and body composition measurements, as well as with biochemical parameters of energy metabolism. Accordingly, alleles and genotypes distribution in a group of 168 young, nonobese Caucasian women measured for chosen body composition parameters, lipid profile, and glucose levels before and after the completion of a 12-week aerobic training program were studied. Although the obtained results showed changes in body mass, BMI, FM, %FM, FFM, TBW, HDL-C, and glucose levels during the training program, none of the examined parameters changed significantly across the *FABP2* genotypes. Instead, we found a main effect of genotype on BMI (*p* = 0.033), with carriers of the Thr54 allele having a higher BMI during the whole study period compared with the Ala54 carriers. We confirm that the *FABP2* Ala54Thr polymorphism may help identify women at risk for overweight and obesity. However, we did not notice evidence of an interaction between physical activity and the Ala54Thr polymorphism on the examined parameters.

## 1. Introduction

Regular physical activity confers many benefits to human health and is a key element of total everyday energy expenditure; as such, it assists in improving body composition and reducing excess body weight. Accordingly, the promotion of exercise, and related exercise training programs, is a key step towards reducing the ever-increasing worldwide epidemic of obesity [1,2]. Li et al., (2010) revealed that physical activity is related with around a 40% reduction in genetic predisposition to overweight and obesity, as assessed by the number of risk alleles carried for genome-wide association studies (GWAS) identified loci [3]. An understanding of the role the various genetic variants exert on the range of the body’s adaptive response to training will support prediction of the consequences of the performed exercises, making training programs more efficient and safer [2]. Over 1000 chromosomal regions and genes have been shown to be involved in body weight and energy metabolism control [4,5]. One of the most promising candidate genetic markers is fatty acid binding protein 2 (*FABP2*), the gene encoding for an intracellular protein which is a member of the FABPs superfamily, acting to bind hydrophobic ligands [6].

FABP2 is a small protein (15 kDa) expressed at high levels in the columnar absorptive epithelial cells of the villi in the intestine (enterocytes). FABP2 includes a single ligand binding site that shows a strong affinity for saturated and unsaturated long-chain fatty acids and is involved in the synthesis of triglyceride-rich lipoproteins. This protein participates in the absorption, intracellular transport, and metabolism of dietary fatty acids and their acyl-CoA esters in small intestine [7,8,9]. There is experimental evidence that high levels of FABP2, expressed in a differentiated enterocyte model, may inhibit fatty acid incorporation by a currently-undefined mechanism [10].

In humans, the *FABP2* gene is located in the long arm of chromosome 4 (4q28-4q31) and consists of 4 exons separated by 3 introns [11]. In 1995, Baier et al. described a common nucleotide transition from guanine (G) to adenine (A) at codon 54 in exon 2 of the *FABP2* gene that results in an alanine (Ala) to a threonine (Thr) change (Ala54Thr; rs1799883) [7]. Many, but not all studies, have shown this amino acid substitution to be a functional mutation which results in physiological consequences at the molecular, cellular, and organ levels [7,9,12].

Although numerous investigators have shown that carriers of the Thr54 variant of *FABP2* have nearly twice the affinity for long-chain fatty acids compared with those with the Ala54 allele, supporting the potential function of the Ala54Thr polymorphism in the etiology of human obesity, others have not [7,13,14,15,16]. A sexual dimorphism with regard to body mass index (BMI) was shown for the Ala54Thr polymorphism [13,14]. The inconsistency of these results may be explained by the fact that in most of the previous studies on physical activity and/or diet composition were usually not taken into account.

In light of the evidence that the missense variation is strongly associated with lipid and carbohydrate metabolism, *FABP2* is an extensively studied candidate gene related to metabolic disorders including obesity, diabetes, and metabolic syndrome [7,9,14,15,16,17]. However, it is unclear whether physical activity levels affect the relationship between the obesity-related traits and genetic variation in *FABP2*. Accordingly, the aim of the present study was to investigate the influence of the Ala54Thr polymorphism on post-training changes of selected body mass and body composition measurements, as well as with biochemical parameters of energy metabolism. We studied the alleles and the genotype distribution in women engaged in a 12-week aerobic training program, searching for any associations.

## 2. Materials and Methods

### 2.1. Ethics Statement

The investigation protocols were performed in accordance with the rules of the World Medical Association Declaration of Helsinki, as well as ethical standards in sport and exercise science research. The procedures were accepted by the Ethics Committee of the Regional Medical Chamber in Szczecin (no. 09/KB/IV/2011 and 01/KB/VI/2017). Participants received a written information sheet concerning the study purpose, procedures used, benefits and risks, as well as a consent form. The experimental protocols were conducted according to the Strengthening the Reporting of Genetic Association studies (STREGA) Statement.

### 2.2. Participants

One hundred sixty eight Polish Caucasian women (age: 21 ± 1 years; body mass: 61 ± 2 kg; body height: 168 ± 2 cm) were included in the study. The following inclusion criteria were considered: low level of physical activity self-reported by each participant with the use of the Global Physical Activity Questionnaire (according to the World Health Organization in the Polish adaptation), no metabolic, neuromuscular or musculoskeletal disorders, refrained from using supplements or medications, nonsmokers. The participants took part in a dietary program and were asked to keep a balanced diet based on their individual dietary plan which was established during a nutritional appointment including a recommendation and a prescription of an adequate diet fitted for individual energy need and nutritional status, as well as a food replacement list. The following average daily macronutrient ratio was recommended (expressed as a percentage of total calories): 45–65% from carbohydrates, 10–20% from protein, and 20–35% from fat (with a simultaneous focus on decreasing the intake of saturated fats and increasing the intake of unsaturated fats). A daily cholesterol intake was recommended to be lower than 300 mg, while the intake of dietary fiber was recommended to be higher than 25 g. All participants kept a daily “diet diary” in which they included everything they ate and drank during the program. The quantity and quality of meals were assessed during diet consultations, which were carried out every week.

### 2.3. Body Composition Measurements

All participants were measured for selected body mass and body composition parameters before and after the 12-week training period. The variables were assessed using the bioimpedance method, using a Tanita TBF 300M electronic scale (Arlington Heights, IL, USA). The following parameters were measured with the electronic scale: total body mass (kg), BMI (kg/m^2^), fat mass (FM, kg), fat mass percentage (%FM, %), fat free mass (FFM, kg), and total body water (TBW, kg) [18].

### 2.4. Biochemical and Hematological Analyses

Fasting blood samples were obtained from the elbow vein in the morning, before the aerobic fitness training program, and after the 36th training unit, which occurred during the 12th week of the program. The biochemical and hematological analyses were performed as described earlier, immediately after blood collection [18]. The parameters received using the Random Access Automatic Biochemical Analyzer for Clinical Chemistry and Turbidimetry A15 (BioSystems S.A., Barcelona, Spain) were: glucose (mg/dL), triglycerides (TGL, mg/dL), total cholesterol (TC, mg/dL), high density lipoprotein cholesterol (HDL-C, mg/dL), and low density lipoprotein cholesterol (LDL-C, mg/dL).

### 2.5. Training Phase

A week-long familiarization stage preceded the proper training stage. During this stage, participants exercised 3 times a week for 30 min at an intensity of about fifty percent of their maximum heart rate (HRmax). After the familiarization stage, the main training program started. Each training unit consisted of the following stages: warm-up routine (10 min), main aerobic routine (43 min), stretching and breathing exercises (7 min). The major aerobic routine consisted of a combination of 2 alternating styles—low and high impact, as presented by Leońska-Duniec et al. [18].

### 2.6. Genetic Analyses

GenElute Mammalian Genomic DNA Miniprep Kit (Sigma, Steinheim, Germany) was used for the extraction of DNA from the buccal cells in accordance with the manufacturer’s procedure. An allelic discrimination assay on a C1000 Touch Thermal Cycler (Bio-Rad, Feldkirchen, Germany) instrument with TaqMan probes was used to genotype all samples in duplicate. TaqMan^®^ Pre-Designed SNP Genotyping Assays (Applied Biosystems, Waltham, MA, USA) (assay ID: C____761961_10), including primers and fluorescently labelled (FAM and VIC) minor groove binder (MGB) probes were used in order to discriminate the *FABP2* Ala54Thr alleles.

### 2.7. Statistical Analyses

Gene counting was used to determine allele frequencies. A chi-square test was used to test the Hardy–Weinberg equilibrium. 2 × 2 mixed-design ANOVA tests were used to test the influence of the Ala54Thr polymorphism of the *FABP2* gene on training response. In addition, the Kolmogorov–Smirnov test was used to check for data normality, as well as the post hoc Fisher’s least significant difference (LSD) test. The models of inheritance, i.e., codominant, dominant, recessive and overdominant were constructed assuming a minor allele as the risk allele. The level of statistical significance was set at *p* < 0.05.

## 3. Results

Genotype frequencies of the *FABP2* polymorphism: Ala54/Ala54—80 (47.62%), Ala54/Thr54—80 (47.62%) and Thr54/Thr54—8 (4.76%) deviated significantly from the Hardy–Weinberg expectations (χ^2^ = 4.67, *p* = 0.031). The selected body composition and metabolic parameters before the 12-week training period initialization are presented in Table 1.

Pre- and post-training body composition and metabolic parameters with respect to the *FABP2* genotype are presented in Table 2. Most parameters (body mass, BMI, %FM, FM, FFM, TBW, HDL-C, and glucose) changed significantly during the intervention (main effect of training), however, the training response did not tend to be modulated by genotype (non-significant genotype × training interactions).

Instead, we found a main effect of genotype on BMI (F(1,166) = 4.63, *p* = 0.033, η^2^ = 0.027) only under the dominant model. The Fisher’s least significant difference test was significant at baseline (Ala54/Thr54 + Thr54/Thr54, 21.9 (19.6–24.4) vs Ala54/Ala54, 21.1 (18.9–23.5); *p* = 0.023) and at the end (Ala54/Thr54 + Thr54/Thr54, 21.6 (19.4–24.2) vs Ala54/Ala54, 20.9 (18.8–23.2); *p* = 0.033). No significant associations were found when other underlying genetic models were assumed.

## 4. Discussion

A number of papers focusing on physical activity behavior and exercise intolerance, cardiorespiratory fitness and endurance performance, muscular strength and power, body weight and adiposity, lipid and lipoprotein metabolism, glucose and insulin metabolism, as well as hemodynamic traits, have revealed genetic markers influencing the functional response of human body to regular physical activities [2,19]. One of the proposed genes associated with obesity, and with potential significance on the body’s adaptive response to training in healthy individuals, is *FABP2*.

Although the present study of Caucasian women demonstrated changes of many selected body mass measurements and biochemical parameters of energy metabolism, such as body mass, BMI, FM, FFM, %FM, TBW, HDL-C, and glucose levels during the 12-week training program, none of the selected parameters changed significantly across *FABP2* genotypes (genotype × training interaction). Another important finding of the data is the identification of a statistically significant association between BMI and *FABP2* genotype. Carriers of the Thr54 variant of *FABP2* had a higher BMI during the entire study period compared with the Ala54 carriers, suggesting that the Thr54 allele is a risk allele involved in excess body weight in Caucasian females. Some genotype effects may be evident only in Thr54/Thr54 homozygotes. Unfortunately, we had insufficient Thr54 homozygotes to analyze separately, which may affect our results.

Many studies have generally focused on the role of the intestinal FABP2 in the absorption, transport, and metabolism of saturated and unsaturated long-chain fatty acids [7,8,9]. Darimont et al., (2000) showed that a high level of FABP2 expressed in a differentiated enterocyte model inhibits incorporation of fatty acid, but the mechanism is still unknown [10]. Additionally, animal model experiments revealed that *Fabp2* null (*Fabpi-/-*) mice show changes in body weight and are hyperinsulinemic. Male *Fabpi-/-* mice had higher plasma TGL and body weight regardless of the fat content in their diet. In contrast, female *Fabpi-/-* mice gained less weight in response to a high-fat diet. The studies on *Fabpi-/-* mice and human Ala54Thr polymorphism suggest that FABP2 is not a direct part of fatty acid absorption but may act as a lipid-sensing component of energy homeostasis that modifies body weight gain in a gender-dependent fashion [20]. However, the exact physiological role of the protein has never been revealed clearly.

While the excess absorption of saturated and unsaturated long-chain fatty acids during sedentary state is considered as a risk factor for obesity, in trained participants this condition may give additional advantage for endurance performance, as suggested by Nasibulina et al. [21], who demonstrated that Thr54 allele frequency was significantly higher in elite Russian endurance (50.0%) and combat (46.2%) athletes compared to controls (32.2%). This is in line with a previous study showing that increased availability of free fatty acids, following a high-fat diet, may provide for increased oxidative potential as evidenced by a growth in VO_2_max and a decreased running time in trained runners [22].

The possible relationship between the Ala54Thr polymorphism and BMI, a measure widely accepted as an index for identifying human overweight and obesity, have been widely described; however, the obtained results are so far conflicting and inconclusive [23]. Fisher et al., (2006) showed that German women with the Thr54 allele have higher BMI and suggested that the Thr54 allele is an effect-modifier for BMI in females [13]. These results are supported by Khattab et al., (2017) who indicated that both the Ala54/Thr54 heterozygotes and carriers of the rare Thr54/Thr54 genotype had significantly higher BMI among an Egyptian population [24]. The presence of the Thr54/Thr54 homozygotes was also significantly associated with obesity in a study of 430 Caucasians of Greek ethnic origin. Here, Tavridou et al., (2009) suggested that the *FABP2* Ala54Thr polymorphism may help identify Caucasian participants at risk for obesity [25]. Additionally, our study confirmed this association in Polish women. Conversely, several studies do not support the relationship between the *FABP2* Ala54Thr polymorphism and BMI. A meta-analysis including 27 studies with 10 974 participants revealed a lack of association between the polymorphism and BMI across populations. Zhao et al., (2011) also analyzed subgroups stratified by ethnicity, gender, health condition but again detected no statistically significant differences [26].

Numerous investigators have also looked for associations between the specific *FABP2* genotype and a variety of biochemical parameters [7,16,24,27,28,29]. Previously, Baier et al., (1995) revealed that the Thr54-containing protein may increase absorption and/or processing of fatty acids in the intestine, therefore increasing fat oxidation. This has been demonstrated to decrease insulin action, resulting in insulinemia, along with increased LDL-C, TC, and TGL [7]. In a meta-analysis of 30 studies with 14,401 participants, the Thr54/Thr54 genotype was strongly associated with increased TC and LDL-C levels, as well as decreased HDL-C levels [16]. Khattab et al., (2017) confirmed that carriers of the Thr54/Thr54 genotype had decreased HDL-C concentrations [24]. Conversely, some studies have reported that lipid profiles do not significantly differ based on *FABP2* genotype [27,28]. A study performed on the Finnish population indicated that fasting serum insulin, glucose, lipids and lipoprotein concentrations, BMR, respiratory quotient, glucose and lipid oxidation levels did not vary among the genotypes [28]. Similar to our results, Kops et al., (2017) described that Thr54 allele carriers showed higher BMI, but with similar lipid profiles in both carriers and noncarriers [29]. A study performed on 111 nondiabetic obese participants following an enriched polyunsaturated fatty acids hypocaloric diet, De Luis et al., (2012) reported no differences in basal values of insulin, glucose, TGL, or LDL-C between genotypes. However, participants with the Thr54 allele demonstrated an improvement in TC, LDL-C, and insulin levels following weight loss [27]. Similarly, Martinez-Lopez et al., (2013) showed that dietary modification through limitation of saturated fat intake significantly decreases BMI, waist-to-hip ratio, waist circumference, and CRP in Thr54 allele carriers [17]. We did not confirm the association between the specific *FABP2* genotype and different post-training changes of selected body composition measurements, lipid profile, and glucose levels. Our findings are consistent with Han (2013), who described that 12-week regular aerobic exercise training may beneficially prevent obesity-related traits; however, none of the examined parameters changed significantly across the *FABP2* genotypes [15]. Additionally, fasting blood lipids, such as TC, HDL-C, and TGL levels, were not affected significantly by the related effects of the *FABP2* Ala54Thr genotype and cardiorespiratory fitness in 837 Japanese participants [30].

The gender-specific effects of the functional influence of the *FABP2* Ala54Thr polymorphism may explain these conflicting results [13,31]. Fisher et al., (2006) reported an important association of the polymorphism with higher BMI and decreased risk of type 2 diabetes only in German female participants [13]. Nakanishi et al., (2004) described that, amongst non-obese women, BMI was higher in the Thr54 carriers [31], which is consistent with our findings. Unfortunately, the presented study included only women, because men did not report to our experiment, and, as a result, we could not examine these dependences. Variation in ethnicities, physical activity, diet, methodology, as well as different statistical approaches in respect to risk ratio estimation, such as pooling homozygous and heterozygous individuals, may also affect results. Additionally, dietary lipid intake is a key determinant of blood cholesterol levels; therefore, differences in lipid intake among different cultures may partly explain the inconclusive results obtained in the mentioned studies [30].

## 5. Conclusions

Our results confirm that the *FABP2* Ala54Thr polymorphism may help identify Caucasian participants at risk for obesity. Carriers of the Thr54 variant had a higher BMI during the entire study period compared with the Ala54 carriers, suggesting that the Thr54 allele is a risk allele involved in body weight excess in Caucasian females. However, whilst many body composition parameters, lipid profile, and glucose levels changed significantly during the 12-week training program, we did not find evidence of the association between the *FABP2* Ala54Thr polymorphism and physical activity on the examined parameters.

## Figures and Tables

**Table 1:** Body composition and metabolic parameters before training program initialization.

Parameter	Pre
body mass (kg)	59.2 (55.2–65.5)
BMI (kg/m^2^)	21.3 (19.9–22.9)
%FM (%)	24.3 (20.2–28.1)
FM (kg)	14.5 (11.2–17.4)
FFM (kg)	45.4 (43.6–47.6)
TBW (kg)	33.2 (31.8–35.0)
TC (mg/dL)	167.0 (150.5–184.5)
TGL (mg/dL)	73.0 (60.0–92.0)
HDL-C (mg/dL)	64.4 (55.0–73.9)
LDL-C (mg/dL)	87.3 (71.2–103.9)
Glucose (mg/dL)	80.0 (71.0–85.0)

Median (lower quartile-upper quartile).

**Table 2:** The *FABP2* genotypes and response to training (2-way repeated measures ANOVA).

Parameter	Ala54/Ala54 (*n* = 80)		Ala54/Th54r + Thr54/Thr54 (*n* = 88)		Genotype	Training	Genotype × Training Interaction
	Pre	Post	Pre	Post	*p*	*p*	*p*
body mass (kg)	59.9 ± 7.8	59.0 ± 7.6	61.4 ± 7.5	60.7 ± 7.4	0.166	<0.0001	0.583
BMI (kg/m^2^) *	21.1 (18.9–23.5)	20.9 (18.8–23.2)	21.9 (19.6–24.4)	21.6 (19.4–24.2)	0.033	<0.0001	0.954
%FM (%)	23.3 ± 5.4	21.7 ± 5.7	24.5 ± 5.4	23.4 ± 5.4	0.079	<0.0001	0.128
FM (kg)	14.3 ± 5.1	13.2 ± 5.2	15.4 ± 5.0	14.5 ± 4.9	0.124	<0.0001	0.379
FFM (kg)	45.5 ± 3.2	46.0 ± 3.2	45.9 ± 3.2	46.2 ± 3.3	0.465	0.00002	0.235
TBW (kg)	33.3 ± 2.8	33.8 ± 2.4	33.6 ± 2.4	33.8 ± 2.4	0.589	0.002	0.318
TC (mg/dL)	170 ± 26	166 ± 26	170 ± 23	171 ± 28	0.514	0.380	0.068
TGL (mg/dL) *	75.9 (52.6–109.7)	77.4 (52.1–115.2)	74.1 (52.4–104.9)	79.2 (58.4–107.3)	0.719	0.175	0.847
HDL-C (mg/dL)	63.7 ± 12.1	59.2 ± 12.9	66.6 ± 14.5	63.3 ± 13.9	0.063	0.000005	0.436
LDL-C (mg/dL)	90.2 ± 23.3	89.9 ± 23.5	87.2 ± 20.5	91.3 ± 23.7	0.798	0.218	0.147
Glucose(mg/dL)	78.1 ± 10.2	74.9 ± 9.7	78.3 ± 9.4	76.2 ± 10.5	0.579	0.001	0.498

Mean ± SD; * geometric mean (antiloged mean of the log data), in brackets-antilog of the log mean + log SD and log mean-log SD; BMI—body mass index; %FM—fat mass percentage; FM—fat mass; FFM—fat free mass; TBW—total body water; TC—total cholesterol; TGL—triglycerides; HDL-C—high density lipoprotein cholesterol, LDL-C—low density lipoprotein cholesterol; the level of statistical significance was set at *p* < 0.05.

## Data Availability

The data presented in this study are available on request from the corresponding author. The data are not publicly available due to privacy/ethical restrictions.
